# Case Report: Prism for PTSD in severe traumatic brain injury with psychiatric comorbidities: two cases

**DOI:** 10.3389/fpsyt.2026.1804685

**Published:** 2026-04-30

**Authors:** Diana Ghelber, Tal Harmelech, Aron Tendler

**Affiliations:** 1 Institute for Advanced Psychiatry, Fort Worth, TX, United States; 2 GrayMatters Health, Haifa, Israel

**Keywords:** amygdala, EEG, neurofeedback, post-traumatic stress disorder, psychiatric comorbidities, traumatic brain injury, treatment resistance

## Abstract

**Background:**

Traumatic brain injury (TBI) with post-traumatic stress disorder (PTSD) is treatment-resistant, with conventional psychotherapy showing limited efficacy due to neurocognitive impairments.

**Case Summary:**

We report two patients with severe TBI and psychiatric comorbidities treated with Prism neurofeedback.

**Case 1:**

44-year-old female, 35 years post-childhood TBI, with agoraphobia and hyperacusis, achieved 42% PTSD reduction and substantial functional gains (social reintegration, independent driving) sustained through 4-month follow-up.

**Case 2:**

40-year-old male, 3.5 years post-adult TBI with bipolar II disorder and severe PTSD (PCL-5 = 62), achieved 90% PTSD reduction with complete remission sustained at 1-month follow-up, enabling return to work and family system transformation. Both patients developed personalized regulatory strategies and maintained gains without relapse.

**Conclusions:**

Prism neurofeedback demonstrates clinically meaningful outcomes in severe TBI-PTSD where traditional psychotherapy shows limited efficacy. The intervention’s circumvention of cognitive processing demands may explain the favorable outcomes. Controlled trials are warranted.

## 1 Introduction

Traumatic brain injury (TBI) frequently co-occurs with post-traumatic stress disorder (PTSD) and multiple psychiatric comorbidities, creating a complex neurocognitive and emotional dysregulation that proves treatment-resistant to conventional psychotherapy and pharmacotherapy (1). The combination of structural brain damage, neuroinflammation, and trauma-related amygdala sensitization creates a uniquely difficult clinical scenario (2). This case series presents two patients with severe TBI and psychiatric comorbidities treated with Prism for PTSD (GrayMatters Health™), amygdala EEG-fMRI-Pattern neurofeedback therapy course, demonstrating substantial functional and symptomatic improvement that compare favorably with outcomes reported in the literature for such patients.

## 2 Case 1: 44-year-old female with childhood TBI and complex PTSD

### 2.1 Clinical history and presentation

#### 2.1.1 Initial traumatic injury and acute treatment

A 44-year-old female presents with Post-Traumatic Stress Disorder (PTSD), Traumatic Brain Injury (TBI) sequelae, Attention-Deficit/Hyperactivity Disorder (ADHD), Generalized Anxiety Disorder (GAD), Social Anxiety, Psychogenic Non-Epileptic Seizures (PNES) and Hyperacusis. The patient’s complex neuropsychiatric presentation originated following a severe TBI at age 9 from a swing set accident. The injury was life-threatening; medical records document that the patient “almost died.” The trauma caused massive structural damage; the medical notes state that the patient “lost cerebellum” and that her “brain stem was squished.” Structural damage was primarily right-sided as confirmed on emergency CT imaging.

Following the trauma, the patient sustained a traumatic loss of consciousness of approximately 24 hours. Initial neurophysiological monitoring during this acute phase showed alpha waves on electroencephalography. The severity of the injury resulted in profound acute neurological sequelae including loss of motor function, aphasia and dysphagia. Over the subsequent two-years of intensive rehabilitation the patient relearned fundamental motor, speech, and swallowing functions that had been lost.

#### 2.1.2 TBI severity documentation

Medical records confirm a Glasgow Coma Scale (GCS) score of 8/15 on presentation and loss of consciousness of approximately 24 hours. Emergency CT demonstrated a depressed right occipital skull fracture with right cerebellar hematoma, brainstem and cerebellar edema, and acute obstructive hydrocephalus, necessitating emergency craniotomy. Follow-up MRI showed encephalomalacia and gliosis of the right posterior temporal lobe and right cerebellar hemisphere, consistent with severe TBI.

Following this initial trauma and recovery period, the patient developed seizure-like episodes. From age 12 to 35, the patient was treated with carbamazepine for seizures that were initially characterized as partial complex seizures. In the year prior to psychiatric evaluation (approximately 2015-2016), the patient experienced a significant increase in seizure-like episodes. Neurological workup included admission to a specialized epilepsy monitoring unit in Dallas where a one-week continuous video-EEG study was conducted. This monitoring led to a change in diagnosis: the episodes were reclassified as PNES. Carbamazepine was discontinued in December 2015. At the time of psychiatric evaluation, the patient had been off antiepileptic medication for one year.

#### 2.1.3 Current presentation and pre-treatment symptoms

The patient presented with a complex history of trauma-related sequelae extending from the initial injury 35 years prior. Quality of life was severely restricted by agoraphobia, hyperacusis and social anxiety. Clinical notes describe a patient who was “stuck in her house,” unable to tolerate more than one visitor a day, and reliant on “safe rooms” during environmental triggers like storms. Her symptom profile included severe startle responses, irritability, cognitive fog, and avoidance behaviors (e.g., earplugs, social isolation). The patient demonstrated marked difficulty leaving the house and expressed pervasive concerns about safety, planning exit strategies constantly, and perceiving threats in routine situations. Notably, the patient exhibited anger outbursts that she did not recognize as being related to PTSD. Historical documentation indicated violent behavior toward family members, including pulling her sister’s hair and dragging her without apparent triggers from the age of 12, and later hitting her husband. In earlier adolescence, she appeared to have enjoyed social activities, travel, and meeting people; however, by the time of treatment evaluation, she was isolated and felt that “nothing is safe.

#### 2.1.4 Baseline medications and management

At the time of enrollment in the Prism treatment protocol, the patient was managed with a combination of pharmacological and non-pharmacological interventions. Pharmacotherapy included sertraline 75 mg for depression and anxiety, lorazepam at 0.5–1 tablet daily (with intermittent attempts at tapering), methylphenidate for ADHD symptom management, and clonazepam as needed for acute anxiety episodes. She supplemented her pharmacotherapy with valerian root, melatonin XR, SAM-E, vitamin D3, and zinc. Additionally, she used various over-the-counter sleep aids including diphenhydramine, general sleep products, and Nyquil to manage her sleep disturbances. The patient was unemployed due to the chronic sequelae of her injury and was unable to drive or shop independently. She experienced severe distress from auditory stimuli such as AC unit noise, storms, and loud voices. Her baseline sleep pattern was excessive, ranging from 10–14 hours per day, and she demonstrated hypersensitivity to sounds and colors that frequently triggered her avoidance and isolation behaviors.

Prior Treatment History: Prior to initiating Prism, the patient had not received any PTSD-targeted treatment. No trauma-focused psychotherapy (e.g., prolonged exposure therapy [PE], cognitive processing therapy [CPT], or eye movement desensitization and reprocessing) had been attempted. Pharmacotherapy had been stable since 2017 (sertraline, lorazepam, clonazepam) with no PTSD-specific additions.

#### 2.1.5 Diagnostic assessment

PTSD diagnosis was established via DSM-5 diagnostic criteria assessed through a structured clinical interview by the treating psychiatrist [DG]. PTSD was diagnosed via comprehensive clinical interview with assessment of DSM-5 symptom domains (re-experiencing, avoidance, and hyperarousal). The PCL-5 was anchored to the childhood TBI. Ongoing symptom monitoring was conducted using the PCL-5, a validated self-report measure of PTSD symptom severity (range 0-80; ≥31 indicates probable PTSD).

### 2.2 Therapeutic intervention: prism for PTSD

In May 2025, the patient consented to treatment with Prism for PTSD using EEG-based neurofeedback to learn to downregulate amygdala-derived biomarker hyperactivity that is elevated in chronic and complex post-traumatic stress disorder.

#### 2.2.1 Treatment course

15 sessions administered twice weekly from May 13, 2025, to July 22, 2025.

Session Structure: Each session lasted approximately one hour, comprising fifteen minutes of EEG setup and strategy planning, thirty minutes of active neurofeedback across five three-minute cycles, and fifteen minutes of debriefing.

#### 2.2.2 Methodology

The patient engaged in an interactive audiovisual interface controlled by her brain activity. She tested multiple mental strategies (e.g., “visualize a rock,” “music,” “reciting text”) to identify which best modulated the signal. Notably, during the first session, the patient experienced a panic attack triggered by an announcer’s voice in the train station simulation, requiring the cycle to be aborted and restarted.

#### 2.2.3 Prism system technical parameters

Prism for PTSD (GrayMatters Health) employs a personalized EEG-based neurofeedback protocol centered on the fMRI-EEG Pattern (EFP) biomarker – a patient-generic index of amygdala activity derived from simultaneous EEG-fMRI co-registration recordings, embedded into the device as a machine learning model that predicts amygdala BOLD activity from scalp EEG in real-time, eliminating the need for fMRI during treatment (3).

The standard treatment course consists of 15 sessions of approximately one hour each, delivered twice weekly over eight weeks, comprising five three-minute feedback cycles per session (75 cycles total) (4). Within each cycle, the patient wears an 8-electrode EEG headset and views a real-time audiovisual scene and uses self-selected mental strategies to downregulate the EFP signal; feedback is delivered through both visual and auditory channels. EEG acquisition and preprocessing followed the manufacturer’s protocol (4).

#### 2.2.4 Selected strategy

The patient adopted a strategy termed “Pulse Points” (sometimes accompanied by music or singing), which consistently yielded high neurofeedback scores (e.g., reaching cycle scores of 181-187). This cognitive strategy involved focusing on physical pulse sensations to induce a state of regulation. The patient brought calming tea containing catnip to multiple sessions, which appeared to support her engagement with the protocol.

### 2.3 Clinical progression and quantitative outcomes

Throughout the 15-session course, the patient completed standardized assessments including the PCL-5 (PTSD), GAD-7 (Anxiety), PHQ-9 (Depression), and Q-LES-Q-SF (Quality of Life). The data reveals a robust downward trend in symptom severity and a correlative rise in quality of life (see **Table 1**).

**Table 1:** Case 1 (female patient) - clinical progression and quantitative outcomes.

Assessment	Early treatment (Sessions 1-3)	Mid-treatment (Sessions 7-10)	End of treatment (Sessions 13-15)	Clinical interpretation
PCL-5 (PTSD)	**38** (probable clinically significant PTSD)	**20-23**	22 (subthreshold/mild PTSD)	**~42% Reduction:** Moved from symptomatic threshold to sub-threshold/mild range.
GAD-7 (Anxiety)	**11** (Moderate)	**6-12**	2 (Minimal)	**~54% Reduction:** Shifted from moderate anxiety to mild/minimal anxiety.
PHQ-9 (Depression)	**14** (Moderate)	**7-10**	5 (Mild)	**~50% Reduction:** Symptoms decreased from moderate to mild.
Q-LES-Q-SF	**40(markedly reduced life satisfaction)**	**43-48**	57(substantial improvement toward normal quality−of−life levels)	**Improvement:** Reported satisfaction with life activities increased significantly.

**Table 1**. Clinical progression and quantitative outcomes for Case 1 (44-year-old female with childhood TBI) throughout 15-session Prism neurofeedback course. PCL-5, PTSD Checklist for DSM-5 (score range 0-80, ≥31 indicates probable PTSD); GAD-7, Generalized Anxiety Disorder-7 (0-21, with 0–4 minimal, 5–9 mild, 10–14 moderate, 15–21 severe); PHQ-9, Patient Health Questionnaire-9 (0-27, with 0–4 minimal, 5–9 mild, 10–14 moderate, 15+ severe); Q-LES-Q-SF, Quality of Life Enjoyment and Satisfaction Questionnaire-Short Form (0-100%, higher scores indicate greater life satisfaction).

### 2.4 Functional impact and long-term follow-up

The quantitative improvements were mirrored by profound functional changes documented in session notes, particularly in her response to major life stressors during the treatment period (see **Table 2** for full episode-of-care timeline).

**Table 2:** Episode-of-care timeline for case 1.

Timepoint	Event	Outcome/Notes
February 1991 (Age 9)	Swing set accident; severe TBI. Emergency craniotomy, partial right cerebellar hemispherectomy.	GCS 8/15; LOC ~24 hours; right cerebellar hematoma, brainstem edema on CT
1991–1993	Intensive rehabilitation; relearned motor, speech, and swallowing functions	Persistent right-sided motor/sensory deficits; intellectual functioning in average range (5-month evaluation)
Age 12–35	Carbamazepine prescribed for seizure-like episodes	Episodes later reclassified as PNES
~2015–2016	Epilepsy monitoring unit admission; 1-week video-EEG	Diagnosis changed to PNES; carbamazepine discontinued December 2015
~2024	Psychiatric evaluation	PTSD, ADHD, GAD, Social Anxiety, PNES, Hyperacusis diagnosed; severe functional impairment
May 13, 2025	Session 1 — Prism for PTSD initiated	Adverse event: panic attack during Session 1; cycle restarted; treatment continued
May–July 2025 (Sessions 1–15)	Active Prism neurofeedback treatment (twice weekly)	Progressive PCL-5 reduction; “Pulse Points” strategy adopted
July 22, 2025	Session 15 — End of treatment	PCL-5 below diagnostic threshold; 42% symptom reduction
September 29, 2025	Bereavement — loss of “Papa Willie”	Utilized neurofeedback strategies successfully; no relapse
4-month follow-up	Follow-up assessment	Sustained gains; independent driving and social reintegration maintained

Key Trends:.

Rapid Response: Significant symptom reduction was noted as early as Session 6-7, with the patient reporting she felt “revitalized” and capable of managing stressors that previously incapacitated her.

Strategy Efficacy: The “Pulse Points” strategy proved highly effective. By Session 14, the patient reported the strategy had become automatic.

Stress Management During Family Crisis: Around Session 4 (May 27, 2025), the patient’s uncle suffered a heart attack over the weekend. Instead of experiencing debilitating distress, the patient reported she was able to use her “Pulse Points” strategy while walking up and down the hospital corridor. She expressed amazement at her ability to maintain this regulation while mobile in a high-stress environment.

#### 2.4.1 Social reintegration

Pre-treatment isolation (maximum 1 visitor/day, avoided crowds) transformed to attending a 4-hour brunch with 9 people, church services, family dinners with loud children, and tolerating crowded waiting rooms.

#### 2.4.2 Auditory processing improvements

Pre-treatment hyperacusis requiring heavy-duty earplugs and retreat to “safe rooms” evolved to watching storms using her strategy, remaining unbothered by AC repair noise, and transitioning to lower-decibel earplugs. She achieved the notable accomplishment of driving a tractor for the first time.

#### 2.4.3 Independence & mobility

From complete dependence on her husband for transport and inability to run errands, the patient resumed driving independently, shopped at multiple stores alone, and engaged in physical labor.

#### 2.4.4 Medication trajectory

Documentation indicates a reduced reliance on rescue medications (Lorazepam, Clonazepam) during the treatment course, with the patient frequently forgetting doses or using them only for acute events rather than daily management.

#### 2.4.5 Patient perspective - grief and bereavement response (September 29, 2025)

Following the loss of “Papa Willie,” a cherished non-biological father figure, the patient developed a sophisticated grief coping strategy of “grieving in small doses” to avoid emotional overwhelm. She remained compliant with medications, denied suicidal ideation, and successfully used her neurofeedback techniques during the funeral despite historical patterns of combative responses to emotional pain. She demonstrated clear understanding that her grief represented a personalized, compartmentalized experience rather than treatment failure.

#### 2.4.6 Adverse events

One adverse event was documented. During Session 1, the patient experienced a panic attack triggered by an announcer’s voice in the train station simulation; the cycle was aborted and restarted without further incident. No additional adverse events or treatment discontinuations occurred across the 15-session course.

## 3 Case 2: 40-year-old male with military TBI, bipolar II disorder, and combat PTSD

### 3.1 Clinical history and presentation

#### 3.1.1 Developmental and military background

A 40-year-old male presents with Bipolar II Disorder, Post-Traumatic Stress Disorder, Attention-Deficit/Hyperactivity Disorder, Generalized Anxiety Disorder, and Traumatic Brain Injury sequelae. His parents divorced when he was just two years old, placing him in a fragmented family system with 12 siblings from multiple marriages. His stepfather, who became his primary male role model, subjected the family to toxic masculinity and verbal abuse that he later described as “like a knife.” This environment forged an angry child who carried a chip on his shoulder throughout his formative years. While he excelled academically and athletically at school, earning sports awards, he remained socially isolated and bullied, developing low confidence and a constant need to prove himself.

At 18, seeking structure and escape, he enlisted in the Marine Corps. Over eight years of service, he deployed twice to Iraq. Though his role was logistical rather than direct combat, he experienced near-miss explosive events, including a car bombing where he witnessed casualties. Upon returning home, he developed hypervigilance, waking in the night to check that everyone was safe, but received no mental health treatment. He was honorably discharged with VA disability for physical injuries only.

The post-military period brought occupational instability and emerging psychological symptoms. He opened a coaching center that failed, briefly taught, then discovered sports psychology. He married and had children—a 17-year-old daughter from a previous relationship and two sons with his wife. His anger issues intensified, with episodes of yelling and slamming doors that mirrored his stepfather’s behavior. He completed two Ironman triathlons, suggesting remarkable physical resilience despite underlying mood instability.

#### 3.1.2 Traumatic brain injury and psychiatric decompensation

At age 37, during Ironman training, he suffered a bicycle accident that caused a severe traumatic brain injury. He spent six days in the ICU, required a craniotomy for a brain hemorrhage, and sustained frontal lobe bruising. The injury left him with anterograde amnesia for the event itself and marked the beginning of a profound decline. Neuropsychological testing were completed followed by neurocognitive rehabilitation, but cognitive deficits in attention, memory, and concentration became evident.

The three years following his TBI (2021-2024) exhibited progressive decompensation across all life domains. He went on medical leave from work for 1.5 years. Multiple surgeries compounded the challenges of his recovery: rotator cuff repair, tympanoplasty and procedures for nose, collarbone, and eyes. His cannabis use escalated as a coping mechanism until he discontinued it in August 2024. Psychiatric symptoms worsened dramatically-depressed mood, restlessness, loss of energy, decreased interest, fatigue, guilt, worthlessness, and irritability. His verbal abuse toward family, which previously occurred mainly once a year, became more frequent. He attempted to stop his psychiatric medications without medical supervision, triggering hypomanic episodes with severe irritability.

#### 3.1.3 Baseline clinical assessment

By September 18, 2024, at age 40, his condition required formal psychiatric intervention. His comprehensive evaluation yielded a complex diagnostic profile: Bipolar II Disorder, Post-Traumatic Stress Disorder, Attention-Deficit/Hyperactivity Disorder, Generalized Anxiety Disorder, and Traumatic Brain Injury.

His baseline assessment on December 4, 2024, revealed a PCL-5 score of 62 (severe PTSD), with GAD-7 of 13 (moderate anxiety). His medication regimen included divalproex sodium for mood stabilization, escitalopram for depression, mixed amphetamine salts for ADHD, and baclofen for bruxism.

#### 3.1.4 Prior treatment history

Prior to initiating Prism, the patient had not received any PTSD=targeted treatment. No trauma-focused psychotherapy had been attempted, and pharmacotherapy was directed toward mood disorder management (divalproex, escitalopram) rather than PTSD. The patient had an established therapist and had participated in individual cognitive behavioral therapy and couples therapy but had not engaged in any PTSD-specific psychotherapy.

#### 3.1.5 TBI severity documentation

The bicycle accident at age 37 resulted in a severe TBI as evidenced by ICU admission (six days), craniotomy for brain hemorrhage evacuation, and frontal lobe contusion. The patient was found unconscious at the scene. Based on clinical documentation by the treating psychiatrist, GCS on presentation was 3/15 and duration of loss of consciousness was 6 days. Neuropsychological testing was completed following the injury, with cognitive deficits in attention, memory, and concentration becoming evident.

#### 3.1.6 Diagnostic assessments

PTSD diagnosis was established by the treating psychiatrist (DG) based on DSM-5 criteria following formal psychiatric evaluation on September 18, 2024. PTSD was diagnosed via comprehensive clinical interview with assessment of DSM-5 symptom domains (re-experiencing, avoidance, and hyperarousal). The PCL-5 was anchored to the 2021 bicycle accident. Baseline PCL-5 score of 62 indicates severe PTSD symptom burden at treatment initiation.

### 3.2 Therapeutic intervention: prism for PTSD

#### 3.2.1 Patient perspective

He began Prism for PTSD treatment on December 4, 2024. Over 15 sessions spanning 10 weeks, he developed personalized strategies rooted in music and meaningful memories: “Pursuit of Happiness,” “Rockstar,” and his wedding day. His evolution was striking—from arriving frazzled, serious, and non-emotive to consistently displaying good affect and smiling.

Treatment timeline: December 4, 2024 – February 5, 2025.

#### 3.2.2 Prism system technical parameters

The same Prism for PTSD system and protocol parameters described for Case 1 (above) were used in Case 2 without modification. Session structure (15 sessions, five three-minute cycles per session, approximately one hour each) and EFP biomarker algorithm were identical (4). The personalized regulatory strategies differed (music- and memory-based in Case 2 *vs*. somatosensory in Case 1), consistent with the adaptive, patient-specific nature of the Prism protocol.

### 3.3 Clinical progression and quantitative outcomes

### 3.4 Functional impact and long-term follow-up

#### 3.4.1 Early post-treatment (March 7, 2025–1 month follow-up)

By March 7, 2025, his PCL-5 remained at 7 with minimal anxiety, demonstrating sustained improvement (see **Table 3**). His family system transformation became evident through improved communication and positive behavioral changes (see **Table 4** for full episode-of-care timeline).

**Table 3:** Clinical outcomes for Case 2 (40-year-old male with adult-onset TBI and bipolar II disorder) from baseline through end of 15-session Prism neurofeedback course.

Assessment	Baseline (Dec 4, 2024)	End of treatment (Feb 5, 2025)	Reduction	Clinical interpretation
PCL-5 (PTSD)	**62** (Severe)	**6**	**90% Reduction**	Moved from severe PTSD to minimal range. Substantial symptom remission.
GAD-7 (Anxiety)	**13** (Moderate)	**7**	**46% Reduction**	Shifted from moderate to mild/minimal anxiety.
PHQ-9 (Depression)	4	**0**	**No change**	No depressive symptoms reported

See **Table 1** caption for scale abbreviations and score interpretations.

**Table 4:** Episode-of-care timeline for Case 2.

Timepoint	Event	Outcome/Notes
~2003–2011 (Age 18–26)	Marine Corps service; two Iraq deployments	Near-miss explosions; witnessed casualties; hypervigilance post-discharge; no mental health treatment
2021 (Age 37)	Bicycle accident during Ironman training; severe TBI. ICU (6 days); craniotomy for brain hemorrhage.	GCS 3/15; LOC 6 days; frontal lobe contusion; anterograde amnesia
2021–2024	Neuropsychological testing; neurocognitive rehabilitation; medical leave 1.5 years	Cognitive deficits in attention, memory, concentration; progressive psychiatric decompensation
September 18, 2024	Formal psychiatric evaluation	Diagnoses: Bipolar II Disorder, PTSD, ADHD, GAD, TBI sequelae
December 4, 2024	Baseline assessment; Session 1 — Prism for PTSD initiated	PCL-5 = 62 (severe); GAD-7 = 13 (moderate)
Dec 2024 – Feb 2025 (Sessions 1–15)	Active Prism neurofeedback treatment (10 weeks)	Progressive symptom reduction; music-based regulatory strategies developed
January 10, 2025	Opened private practice mid-treatment	Patient-initiated occupational restoration
February 5, 2025	Session 15 — End of treatment	PCL-5 = 6; 90% PTSD symptom reduction; depressive remission
March 7, 2025 (1-month follow-up)	Follow-up assessment	PCL-5 = 7; sustained remission; family system improvements

Key Achievements:.

90% PTSD reduction compares favorably with outcomes reported in the literature for TBI-PTSD.

Complete remission of depressive symptoms by end of treatment.

Occupational restoration: Opened private practice on January 10, 2025.

Adverse Events: No adverse events were reported during the 15-session treatment course. The patient tolerated all sessions without discontinuation.

Comparative Analysis.

Case 1 *vs*. Case 2. For comparison of clinical characteristics, interventions, and outcomes between Case 1 (childhood-onset TBI) and Case 2 (adult-onset TBI) see **Table 5**.

**Table 5:** Comparative analysis of clinical characteristics, interventions, and outcomes between Case 1 (childhood-onset TBI) and Case 2 (adult-onset TBI).

Dimension	Case 1 (Female, Childhood TBI)	Case 2 (Male, Adult TBI)
Age at TBI	9 years	37 years
TBI Mechanism	Playground accident	Bicycle accident (Ironman training)
Time Post-TBI at Treatment	35 years	~3.5 years
Primary Psychiatric Comorbidities	PTSD, ADHD, GAD, Social Anxiety, Hyperacusis	Bipolar II, PTSD, ADHD, GAD
Baseline PCL-5	38	62
PCL-5 Reduction	~42% (to 22)	**90% (to 6)**
Treatment Duration	15 sessions, 9 weeks	15 sessions, 10 weeks
Selected Strategy	“Pulse Points” (somatosensory)	Music-based (“Pursuit of Happiness,” “Rockstar”)
Functional Outcome Focus	Social reintegration, auditory processing	Occupational restoration, family communication
Medication Reduction	Reduced benzodiazepine use	Maintained medication compliance
Follow-Up Stability	Sustained gains through Sept 2025 (4.5 months and continuing)	Complete remission maintained through March 2025 (1 month)

Comparison with Evidence-Based Treatments: Approaches currently available to treat cases of Traumatic Brain Injury with PTSD include psychotherapy and pharmacotherapy.

#### 3.4.2 Psychotherapy

While prolonged exposure (PE) and cognitive processing therapy (CPT) demonstrate efficacy in treating PTSD among patients with TBI history, important limitations exist (5). Sleep disturbances frequently persist after trauma-focused therapy despite improvements in daytime PTSD symptoms, with the majority of patients continuing to experience clinically significant insomnia and nightmares following PE treatment (6).

CPT augmented with Cognitive Symptom Management and Rehabilitation Therapy (CogSMART) shows promise in addressing both PTSD and cognitive symptoms in TBI-PTSD populations, with SMART-CPT demonstrating additional benefits for attention, verbal learning, and problem-solving compared to CPT alone (7). However, baseline verbal memory performance strongly predicts treatment response to trauma-focused psychotherapy (8–10), and patients with poorer executive functioning show increased dropout rates and reduced treatment gains (11).

Critically, both PE and CPT require cognitive engagement with trauma narratives—processes potentially impaired by TBI-related deficits in attention, memory, and executive function. PRISM employs real-time EEG pattern recognition targeting amygdala-derived dysregulation through personalized sensory modulation, bypassing the need for verbal trauma processing that may be compromised in severe TBI populations.

#### 3.4.3 Medications

SSRIs and SNRIs show efficacy for PTSD but demonstrate unclear benefit for TBI-related cognitive deficits (12).

Anticonvulsants (e.g., valproate) demonstrate mixed findings for PTSD and primarily serve mood stabilization (12).

Stimulants (e.g., methylphenidate, mixed amphetamine salts) improve attention in TBI but may worsen PTSD hyperarousal symptoms (13).

Benzodiazepines are generally contraindicated due to cognitive impairment risk and fall hazard in TBI populations (14).

#### 3.4.4 Mechanistic considerations

Unlike PE and CPT, which require cognitive engagement with trauma narratives—processes impaired by TBI-related attention, memory, and executive dysfunction—Prism employs real-time EEG pattern recognition targeting limbic dysregulation directly. By using personalized sensory strategies (somatosensory in Case 1, music-based in Case 2) to modulate emotional circuits without explicit recall demands, Prism bypasses the neurocognitive barriers that constrain traditional psychotherapy.

Typical learning strategies that are utilized in TBI-PTSD psychotherapy require the engagement of multiple brain regions. The repeated processing of detailed memories and emotions, with current emotions is challenging for a brain with intact functioning. It is even more difficult for a brain with diffuse axonal injuries.

The Prism for PTSD system allows the patient to engage with the same exact simple scenario repeatedly over three-minute cycles. A typical treatment course of fifteen treatment sessions presents the same scenario seventy-five times. The patient will try different mental strategies, to change the interface, calming a busy noisy waiting room or train station. This simplifies the learning process.

Not surprisingly, the same strategy that works to down regulate the amygdala network for the interface works on daily stressors that trigger PTSD symptoms. After the full-time course, symptom severity across clusters showed marked reduction without having challenged the injured brain to process traumas while they were still suffering from PTSD.

#### 3.4.5 Extended neuroplastic window

The traditional model posits optimal TBI recovery within 1–2 years post-injury (15). Case 1 demonstrates substantial recovery 35 years post-injury, while Case 2 shows 90% symptom reduction 3.5 years post-injury—timeline that may be inconsistent with passive natural recovery alone. These findings are consistent with the hypothesis that Prism facilitates connectivity restoration in chronically dysregulated networks, potentially extending the neuroplastic window; controlled studies are needed to confirm this.

#### 3.4.6 Neurophysiological markers of engagement

Both patients reported altered somatic sensations during focused regulatory states (tingling, bodily awareness intensification) - possibly consistent with prefrontal regulatory network engagement, though the neurophysiological basis of these subjective somatic experiences warrants further investigation.

#### 3.4.7 Clinical and research implications

Treatment-Refractory Population: Prism may serve patients who fail standard approaches. The magnitude of improvement in severe TBI with multiple psychiatric comorbidities suggests circuit-based targeting may overcome multisystem barriers that limit psychotherapy and pharmacotherapy efficacy.

Heterogeneous Strategy Selection: The divergent strategy choices (somatosensory *vs*. music-based) suggest Prism’s adaptive interface accommodates individual neurophysiological and cognitive profiles. Case 1’s somatosensory focus may reflect heightened bodily awareness post-TBI, while Case 2’s music-based approach leveraged preserved reward circuitry.

Our cases demonstrated reduced rescue medication use or maintained compliance without worsening, suggesting Prism may reduce polypharmacy burden in complex TBI-PTSD.

Case 1 sustained gains through 4-month follow-up and Case 2 demonstrated sustained remission at 1-month follow-up, contrasting with relapse rates in traditional psychotherapy literature.

#### 3.4.8 Limitations and confounds

Interpretation of these findings must consider multiple confounds inherent to uncontrolled case reports, including natural symptom fluctuation, regressing to the mean, expectancy effects, nonspecific therapeutic contact, medication stabilization, and concurrent psychosocial support. Therefore, improvement cannot be attributed solely to the Prism intervention. These findings should be interpreted as hypothesis-generating; controlled trials with sham or yoked-feedback are needed to establish both efficacy and the specific contribution of the EFP signal. Assessment relied on the PCL-5 self-report instrument; future studies should include clinician-administered measures such as the CAPS-5 for more rigorous diagnostic confirmation.

## 4 Conclusion

This case series suggests that Prism for PTSD offers a promising alternative for treatment-resistant TBI-associated psychiatric comorbidities. By targeting amygdala-derived dysregulation through personalized sensory modulation without requiring cognitively demanding trauma narrative processing, Prism was associated with substantial clinical gains in both childhood-onset and adult-onset severe TBI populations. The 42-90% PTSD symptom reductions resulted in both patients no longer meeting diagnostic criteria for PTSD at end of treatment (PCL-5 <31), a magnitude of improvement that is notable given the severity and chronicity of these cases and the known barriers to treatment response in TBI-PTSD (16). Prospective trials with advanced imaging and detailed assessments are warranted to establish Prism’s position in the treatment hierarchy for this profoundly challenging and underserved population.

## Data Availability

The original contributions presented in the study are included in the article/supplementary material. Further inquiries can be directed to the corresponding author.
